# Preliminary comparative study of cortical thickness in HIV-infected patients with and without working memory deficit

**DOI:** 10.1371/journal.pone.0261208

**Published:** 2021-12-10

**Authors:** Rafael Ferracini Cabral, Diogo Goulart Corrêa, Nicolle Zimmermann, Gustavo Tukamoto, Tadeu Takao Almodovar Kubo, Rochele Paz Fonseca, Marcos Martins Silva, Nina Ventura Wilner, Paulo Roberto Valle Bahia, Emerson Leandro Gasparetto, Edson Marchiori

**Affiliations:** 1 Department of Radiology, Hospital Universitário Clementino Fraga Filho, Federal University of Rio de Janeiro, Rio de Janeiro, Rio de Janeiro, Brazil; 2 Department of Radiology, Clínica de Diagnóstico por Imagem—Diagnósticos da America (CDPI-DASA), Rio de Janeiro, Rio de Janeiro, Brazil; 3 Department of Radiology, Paulo Niemeyer State Brain Institute, Rio de Janeiro, Rio de Janeiro, Brazil; 4 Department of Psychology, Paulo Niemeyer State Brain Institute, Rio de Janeiro, Rio de Janeiro, Brazil; 5 Department of Psychology, Pontifical Catholic University of Rio Grande do Sul, Porto Alegre, Rio Grande do Sul, Brazil; 6 Department of Neurology, Hospital Universitário Clementino Fraga Filho, Federal University of Rio de Janeiro, Rio de Janeiro, Rio de Janeiro, Brazil; The University of New South Wales, Neuroscience Research Australia, AUSTRALIA

## Abstract

**Purpose:**

Changes in cerebral cortical regions occur in HIV-infected patients, even in those with mild neurocognitive disorders. Working memory / attention is one of the most affected cognitive domain in these patients, worsening their quality of life. Our objective was to assess whether cortical thickness differs between HIV-infected patients with and without working memory deficit.

**Methods:**

Forty-one adult HIV-infected patients with and without working memory deficit were imaged on a 1.5 T scanner. Working memory deficit was classified by composite *Z* scores for performance on the Digits and Letter-Number Sequencing subtests of the Wechsler Adult Intelligence Scale (third edition; WAIS-III). Cortical thickness was determined using FreeSurfer software. Differences in mean cortical thickness between groups, corrected for multiple comparisons using Monte-Carlo simulation, were examined using the query design estimate contrast tool of the FreeSurfer software.

**Results:**

Greater cortical thickness in left *pars opercularis* of the inferior frontal gyrus, and rostral and caudal portions of the left middle frontal gyrus (cluster 1; *p* = .004), and left superior frontal gyrus (cluster 2; *p* = .004) was observed in HIV-infected patients with working memory deficit compared with those without such deficit. Negative correlations were found between WAIS-III–based *Z* scores and cortical thickness in the two clusters (cluster 1: *ρ* = –0.59; cluster 2: *ρ* = –0.47).

**Conclusion:**

HIV-infected patients with working memory deficit have regions of greater thickness in the left frontal cortices compared with those without such deficit, which may reflect increased synaptic contacts and/or an inflammatory response related to the damage caused by HIV infection.

## Introduction

More than 1 million individuals in the United States and more than 40 million people worldwide are currently infected with the human immunodeficiency virus (HIV) [1]. The advent of highly active antiretroviral therapy (HAART) has markedly reduced HIV-associated mortality, but no comparable reduction of neurological complications has been achieved [2]. Despite a decline in the incidence of HIV-associated dementia, the prevalence of milder HIV-associated neurocognitive disorders (HAND) remains unchanged [3]. Central nervous system (CNS) involvement persists, probably due to the persistence of HIV reservoirs in the brain, despite blood viral suppression and immune reconstitution [4].

The definitive diagnosis of HAND, preferably using standardized neuropsychological tests, is based on evaluation of the following cognitive domains: working memory / attention, inhibition/cognitive flexibility, memory (learning and recall), speed of information processing, sensory-perceptual and motor skills, and verbal language [3]. The main cognitive domains affected in HAND are visual and verbal working memory and attention [5, 6]. Working memory, defined as the ability to retain and manipulate information for short periods of time is required for the maintenance of awareness and concentration, and is very important for executive functions and learning. Although HAART generally improves neuropsychological function in HIV-infected patients, it does not improve working memory deficit, and may even exacerbate it with ongoing infection [7]. Working memory deficit in HIV-infected patients is related to poorer medication adherence, self-reported cognitive complaints [8], and dependence in activities of daily living [9]. Moreover, patients with HAND of all stages have an increased mortality risk [10].

Many neuroimaging studies have demonstrated the occurrence of volumetric changes in gray and white matter in the brains of HIV-infected patients [11–15]. Specifically, volume reductions in the amygdala, caudate nucleus, thalamus, and hippocampus, as well as in neocortical regions such as the cingulate cortex, have been described [16]. Such cerebral atrophy has been correlated with poor cognitive performance in some studies [15, 16], but not in others [17]. However, most studies of this nature have not involved the consideration of cognitive domains or the inclusion of patients with HIV-associated dementia [12]. A clear understanding of the relationship between cortical thickness and working memory in HIV-infected patients is needed to identify structural brain changes that are involved in neurocognitive deterioration in these patients. Thus, this study was conducted to investigate brain cortical thickness in HIV-infected patients with and without working memory deficit.

## Materials and methods

### Ethics committee approval

This study was approved by the Ethics Committee of the Clementino Fraga Filho University Hospital (CEP151/08), and all participants provided written informed consent prior to inclusion in this study.

### Subjects

Between September 2011 and February 2015, 55 patients with HIV infection for ≥5 years, as confirmed by enzyme-linked immunosorbent assay and western blot, were selected randomly from the hospital’s database. Exclusion criteria were self-reported illicit drug use within the past year (cocaine and crack cocaine, marijuana, hallucinogens and dissociative drugs, synthetic cannabinoids, methylenedioxymethamphetamine (MDMA–ecstasy or molly), methamphetamine, opium, heroin and other opioids), neurological disorder (e.g., current or past CNS infection), psychiatric illness, magnetic resonance (MR) imaging contraindication, and abnormal findings on conventional brain MR imaging sequences. Five patients had to be excluded from the study because they met clinical exclusion criteria or had MR imaging contraindication.

All 50 patients underwent MR imaging and neuropsychological testing. Five were excluded because of MR imaging alterations secondary to previous neurological diseases or opportunistic infection. An additional 4 patients were excluded in the subsequent matching of the two groups by age, years of education, and gender.

All the remaining 41 patients were divided into two groups according to the presence or absence of working memory deficit, based on composite Z scores for the Digits and Letter-Number Sequencing subtests of the Wechsler Adult Intelligence Scale (third edition; WAIS-III) [18].

All HIV-infected patients who participated in this study had undetectable plasma viral loads (below 50 copies of HIV-1 RNA per milliliter of blood) and were receiving HAART. Of those with working memory deficits (*n* = 17; Table 1) 11 patients had Memorial Sloan Kettering (MSK) ratings [3] of 0.0, 3 had ratings of 0.5, and 3 had ratings of 1.0. Of the 24 participants without working memory deficits, 17 had ratings of 0.0, 5 had ratings of 0.5, and 2 had ratings of 1.0. The divisions of groups according to the Fascati criteria [3] show that of the patients with working memory deficit (n = 17), 11 met the definition of HAND, of these 6 were asymptomatic neurocognitive impairment (ANI) and 5 were mild neurocognitive disorder (MND). In the group of patients without working memory deficit (n = 24), 12 met the definition of HAND, of which 10 were ANI and 2 were MND.

**Table 1:** Sociodemographic and clinical data of HIV-infected patients.

	Groups	Mean	Standard deviation	Range^c^	df	t	*p*
Age in years^a^	HIV-infected with working memory deficit	52.53	6.92	(41–62)	39.00-	1.01 -	.32 -
	HIV-infected without working memory deficit	50.33	6.83	(31–65)			
Years of known HIV infection^a^	HIV-infected with working memory deficit	13.26	3.49	(8–21)	39.00-	0.05 -	.96 -
	HIV-infected without working memory deficit	13.17	5.43	(3–23)			
Years on HAART^a^	HIV-infected with working memory deficit	12.21	4.38	(3–18)	39.00 -	0.05 -	.96 -
	HIV-infected without working memory deficit	12.28	5.47	(3–20)			
Years of education^a^	HIV-infected with working memory deficit	8.41	4.30	(3–15)	39.00-	-1.77 -	.14 -
	HIV-infected without working memory deficit	10.63	3.70	(4–20)			
CD4 T Lymphocyte count at the time of MRI (cells/μL)^a^	HIV-infected with working memory deficit	649.65	338.31	(47–1198)	39.00-	-0.32 -	.75 -
	HIV-infected without working memory deficit	685.83	365.03	(138–1819)			
CD4 T Lymphocyte count nadir (cells/μL)^a^	HIV-infected with working memory deficit	208.31	110.62	(16–341)	39.00-	-0.70 -	.49 -
	HIV-infected without working memory deficit	183.09	107.16	(38–480)			
Sex^b^	HIV-infected with working memory deficit	14M/3W	-	-	1.00 -	- -	.40 -
	HIV-infected without working memory deficit	17M/7W	-	-			

M, men; W, women. Statistical analyses used

^a^independent *t* test

^b^chi-squared test

^c^(minimum value and maximum value).

All participants were Brazilian, right-handed and only spoke Portuguese. No significant difference in sex, age, years of education, duration of known HIV infection, years on HAART, or CD4 T lymphocyte count nadir and at the time of MR imaging was found between HIV-infected patients with and without working memory deficit (see Table 1).

### Neuropsychological assessment

Working memory scores were calculated based on WAIS-III tasks [19] and converted to *Z* scores [(raw score–normative mean) / normative standard deviation]. Participants with composite *Z* scores [(*Z* Digit Span + *Z* Letter-Number Sequencing) / 2] ≤ –1.5 were considered to have working memory deficits. The cut-off of –1.5 standard deviations below the normative mean has been used widely to identify clinically relevant deficits and aligns with the diagnostic criteria for mild cognitive impairment [20, 21].

The WAIS-III is used widely to assess intelligence and cognitive components. The Digits and Letter-Number Sequencing tasks were used in this study to assess participants’ working memory. Cunha et al. [22] has reported on the adaptation of the WAIS-III for the Brazilian population, and its validation and normative application in this population.

To avoid bias by other critical cognitive functions involved in working memory ability, as suggested by Antinori et al. [3], we assessed and compared between groups the speed of information processing, sensory-perceptual and motor skills, verbal language, and executive functions domains. Table 2 shows the variables comprising each cognitive domain and the neuropsychological tests used to assess them. Mean *Z* scores showed no significant difference in performance between groups in any domain, except working memory (Mann–Whitney *U* test; Table 3). A neuropsychologist (RPF) with specific training and 8 years of experience in cognitive testing administered all neuropsychological tests on the day of MR imaging examination.

**Table 2:** Cognitive domains with their corresponding neuropsychological tests and specific variables for the composite scores.

Cognitive Domains	Neuropsychological Tests	Variables
Executive functions	Trail making test	Time B and Errors B, B/A Time, B-A time
	Stroop color and word test	
		Time Color-word Page Score, interference score
	Hayling test	
		B/15 errors, B/45 errors, B-A time
Processing speed	Bells cancellation test	Time 1
	Hayling test and trail making test	Times Parts A
Sensory-perceptual and motor skills	Brazilian brief neuropsychological assessment battery NEUPSILIN	Constructive praxis task
Verbal language	Montreal communication evaluation battery	Semantic and phonemic verbal fluency tasks
Working memory	Wechsler adult intelligence scale–III	Digits and letter-number sequencing tasks

**Table 3:** Comparative analysis among groups on cognitive domains *Z* scores.

Cognitive Domains	Groups	Median *Z* Score (IQR)	Range^a^	*U*	*p*
Executive functions	HIV-infected with working memory deficit	-0.81 (-1.87–0.03)	(-3.51–0.56)	124.00	.06
		-0.28 (-0.71–0.06)	(-1.25–1.05)		
	HIV-infected without working memory deficit				
Processing speed	HIV-infected with working memory deficit	-0.99 (-2.26 –-0.59)	(-3.65–0.41)	151.50	.26
		-0.95 (-1.62 –-0.17)	(-2.43–1.02)		
	HIV-infected without working memory deficit				
Sensory-perceptual and motor skills	HIV-infected with working memory deficit	-0.37 (-2.07–0.56)	(-2.62–0.95)	127.50	.36
		-0.06 (-0.98–0.56)	(-3.07–1.93)		
	HIV-infected without working memory deficit				
Verbal language	HIV-infected with working memory deficit	-0.68 (-1.20 –-0.36)	(-2.08 –-0.1)	136.50	.07
		-0.21 (-1.34–0.03)	(-2.1–0.95)		
	HIV-infected without working memory deficit				
Working memory	HIV-infected with working memory deficit	-2.02 (-2.50 –-1.80)	(-3.68 –-1.57)	0.00	< .001
		-0.47 (-1.21–0.12)	(-1.49–2.72)		
	HIV-infected without working memory deficit				

IQR, interquartile range

^a^(minimum value and maximum value).

### MR imaging protocol

MR imaging was performed using a 1.5 T scanner (Avanto; Siemens, Erlangen, Germany) with an eight-channel phased-array head coil. The MR imaging protocol included axial FLAIR; TR, 9000 ms; TE, 83 ms; FOV, 230 mm; matrix, 244 × 256; section thickness, 4.5 mm with a 10% gap; flip angle, 180°; inversion time, 2500 ms and sagittal T1 three-dimensional (3D) MPRAGE–weighted (TR, 2730 ms; TE, 3.26 ms; TI, 1000 ms; FOV, 256 mm; matrix, 192 × 256; section thickness, 1.3 mm; flip angle, 7°; voxel size, 1.0 mm × 1.0 mm × 1.3 mm) sequences. Subject’s heads were stabilized with tape across the forehead and padding around the sides. A neuroradiologist (ELG) with 16 years of experience reviewed the MR images. All images acquired were of sufficient quality for postprocessing.

### Assessment of cortical thickness and statistical analysis

Cortical reconstruction of sagittal T1 3D MPRAGE–weighted images was performed using FreeSurfer (version 5.3.0; http://surfer.nmr.mgh.harvard.edu) as described previously [23, 24]. Briefly, the procedure included motion correction, the removal of nonbrain tissue deformation, Talairach transform computation, correction for signal intensity and automated topology, tessellation of the gray/white matter boundary, and inflation and registration of the cortical surface. This technique uses intensity and continuity information from the 3D image volume to represent cortical thickness, defined as the distance between the gray/white matter boundary and the gray matter/cerebrospinal fluid boundary. Cortical thickness maps were made for each patient, and mean cortical thickness was measured. Cortical thickness was compared between participants with and without working memory deficits, using a cluster-forming threshold of 1.3 (*p* < .05), adopting the query design estimate contrast (QDEC) tool in FreeSurfer [25], application of a smoothing factor of 10, and Monte-Carlo simulation (significance at *p* < .05) to correct for multiple comparisons. FreeSurfer enables the hypothesis-free assessment of differences in cortical thickness between groups.

Correlations of cortical thickness with working memory *Z* scores in areas with significant differences in the whole sample were examined using the Spearman’s rank correlation coefficient.

## Results

**Fig 1:**
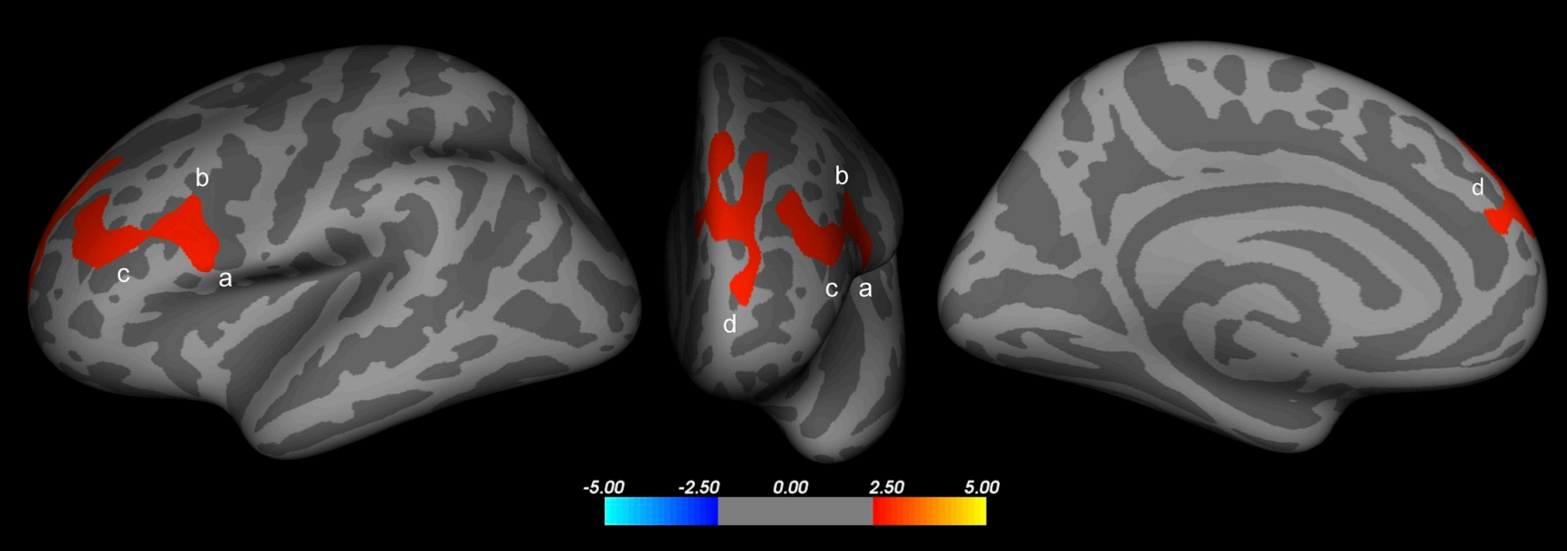
Clusters of significantly greater cortical thickness the left hemisphere in HIV-infected patients with (vs. HIV-infected without) working memory impairment. Left *pars opercularis* of the inferior frontal gyrus (a), caudal (b) and rostral (c) portions of the middle frontal gyrus, and left superior frontal gyrus (d).

**Fig 2:**
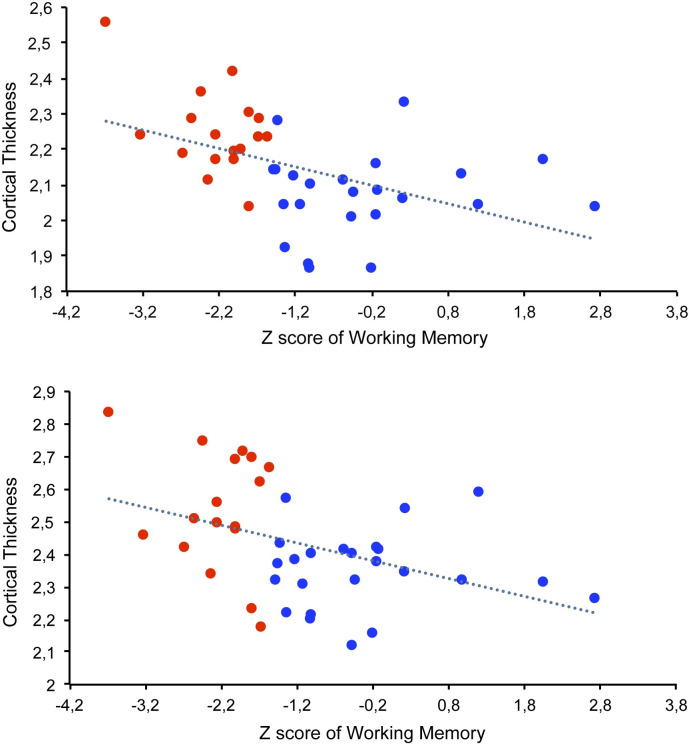
Distribution of cortical thickness (in millimeters) in clusters according to *Z* scores. Clusters 1 (a) and 2 (b) in HIV-infected patients with (red) and without (blue) working memory deficits.

Following the correction for multiple comparisons, adjusted analyses revealed greater cortical thickness in two clusters in HIV-infected patients with working memory deficit compared with those without such deficit (Table 4). Cluster 1 affected areas in the left *pars opercularis* of the inferior frontal gyrus, and the rostral and caudal portions of the left middle frontal gyrus. Cluster 2 affected areas of the left superior frontal gyrus (Fig 1). Negative correlations were found between WAIS-III–based *Z* scores and cortical thickness in the two clusters (cluster 1: *ρ* = –0.59; cluster 2: *ρ* = –0.47; Fig 2). Cortical thickness was not correlated with performance in any other cognitive domain (Table 5).

**Table 4:** Clusters of significant altered cortical thickness between HIV-infected patients groups.

							Talairach coordinates				
Region	Hemisphere	BA	Max-log (p)	VtxMax	Size (mm^2^)	Thickness^a^ (mm)		x	y	z	*p*
Cluster 1	LH	9/10/44/46	3,672	44,468	1166	(1.86749–2.5605)		-36	18	20	.004
Cluster 2	LH	8/9/10	2,849	65,563	1186	(2.21775–2.83781)		-10	45	38	.004

BA, Brodmann`s area; LH, left hemisphere; ^a^average thickness (minimum value and maximum value).

**Table 5:** Correlations between the performance of cognitive domains and the cortical thickness of the clusters.

	Cluster 1		Cluster 2	
Cognitive Domains	*ρ*	*p*	*ρ*	*p*
Executive functions	-0.031	.85	0.032	.85
Processing speed	0.016	.92	-0.039	.81
Sensory-perceptual and motor skills	-0.045	.79	0.034	.84
Verbal language	-0.249	.12	-0.284	.07
Working memory	-0.587	< .001	-0.470	.002

## Discussion and conclusion

This investigation revealed greater thickness of the left frontal cortices in HIV-infected patients with working memory deficit compared with those without such deficit. Cortical thickness in these areas correlated negatively with WAIS-III–based *Z* scores.

Several postmortem studies have demonstrated widespread neuronal loss involving the basal ganglia, the entire cerebral cortex, and brain stem structures in HIV-infected patients [26]. Subcortical gray matter structures are particularly vulnerable to the effects of HIV brain infection [12], but the reason for this preferential involvement remains unknown [27]. The pattern of atrophy observed in cognitively impaired HIV-infected patients involves the nigrostriatal and frontostriatal systems. These changes are consistent with the clinical characteristics of HAND, including the impairment of working memory, executive function, attention, and motor function [28].

Working memory is an essential component of many complex cognitive functions, and it is critically dependent on the integrity of the neural circuitry, including the prefrontal cortex and striatum. A recent meta-analysis of studies of executive function and HIV serostatus [29] suggested that working memory is the most commonly affected “cognitive” component of executive function among HIV-infected individuals. The Digits and Letter-Number Sequencing subtests of the WAIS are used widely to evaluate working memory [30]. We used specific tests to assess the function of cognitive domains other than working memory for group matching, assessing all cognitive domains recommended in the definition of HAND [3]. As no significant difference was observed in other domains, our findings can be attributed to working memory deficit.

Functional MR imaging and positron emission tomography have shown the recruitment of specific brain regions during a working memory task, demonstrating that activation of the prefrontal cortex, parietal regions, cingulate gyrus, and hippocampus is associated with working memory processing, in unimpaired young adults [31]. The greater frontal lobe cortical thickness in HIV-infected patients with working memory deficit in this study is consistent with these findings.

The most common neuroradiological brain abnormalities in HIV-infected patients are nonspecific, consisting of diffuse cerebral atrophy with symmetrical white-matter hyperintensity on T2-weighted and FLAIR sequences, in the absence of contrast enhancement and the mass effect [32]. Thus, many quantitative methods have been used for the early detection of brain abnormalities in HIV-infected patients. Previous studies have shown diffuse brain atrophy and volumetric reduction in specific cortical and subcortical brain structures [11]. Most authors have reported correlations of diffuse or regional atrophy with cognitive impairment [16, 33], motor dysfunction, advanced Centers for Disease Control stages of HIV infection, and longer disease duration [11]. Previous studies of changes in cortical thickness in HIV-infected patients have documented atrophy in the primary sensory and motor cortices, medial frontal and premotor cortices, parietal association cortex [13], posterior and inferior temporal lobes, parietal lobes, cerebellum [15], and temporal and anterior cingulate cortices [12]. Kallianpur et al. [14] found cortical thinning in the bilateral insula, orbitofrontal and temporal cortices, right superior frontal cortex, and right caudal anterior cingulate in patients receiving HAART with detectable HIV DNA in the peripheral blood, compared with patients receiving HAART with no detectable HIV DNA in the peripheral blood. Differently, in the current study, we found areas of greater cortical thickness in patients with cognitive impairment compared to patients without such deficit. This may have happened because we included only patients without dementia. Also, the participating patients with working memory deficits were in early phases of HAND, different from those found in previous studies, which included patients with dementia, possibly reflecting advanced phases of HAND [13–15].

Cortical thinning has been observed in the context of aging and in association with numerous diseases [34]. Areas of increased cortical thickness have been described in individuals with psychiatric disorders, pediatric obstructive sleep apnea and autism, as well as in meditators, drug users, online gamers, and professional athletes [35–37]. Asymptomatic PSEN1 mutation carriers for familial Alzheimer’s disease (AD) presented increased cortical thickness in the precuneus and parietotemporal areas compared to healthy controls, with reduced thickness observed with disease progression [38]. Several evidences in the literature on morphometric studies in humans [39–42], as well as in animal models [43, 44], and pathological data [41, 42] support a possible increase in cortical thickness in early stages of AD. Reactive neuronal hypertrophy and inflammatory response are likely to cause increased cortical thickness in the early and asymptomatic phase of familial AD, while the predominance of neuronal loss occurs in the symptomatic phase of the disease. Similarly, Paulsen et al. [45] found that preclinical Huntington’s disease participants had a significantly higher proportion of cortical gray matter compared with healthy control subjects. These findings may also occur in HIV-infected population, as the greater cortical thickness found in HIV-infected patients with working memory deficit and in early stages of HAND, in the current study, may be related with similar underlying pathological processes, when compared to the early stages of familial AD.

Functional MR imaging studies in HIV-infected patients are able to detect alterations even in the early stages of HAND, which may even precede structural brain lesions detectable by imaging methods [46]. Chang et al. [47] observed significantly greater blood oxygenation level–dependent (BOLD) activation, predominantly in the frontal, inferior lateral prefrontal cortex, and supplementary motor area, during working memory tasks in HIV-infected patients compared with controls. This extension of brain activation is probably related to the saturation of neural activity in normally activated regions and the need to recruit adjacent neural substrate. The frontostriatal system is often most severely affected in patients with HAND, as demonstrated in neuropathological and neuroimaging studies [48, 49], and this damage may necessitate even greater modulation, with recruitment of additional neural processes and greater frontal activation during working memory tasks. Increased activation in selected brain regions also has been reported in patients with other brain disorders, including adults with mild traumatic brain injuries, those with schizophrenia, and those at risk of Alzheimer disease, as well as in children with attention deficit disorder [50]. Thus, MR imaging studies of people with other brain disorders are needed to clarify the concept that neuronal injury leads to increased use of the brain’s reserve capacity.

A magnetoencephalography study of encoding operations and the memory maintenance processes of working memory revealed no difference during the encoding period, but a significantly stronger decrease in alpha activity in the left supramarginal gyrus, areas of the left inferior frontal gyrus (i.e., Broca’s area), and the left cerebellum in HIV-infected patients compared with controls [51]. Combined electroencephalography/fMRI studies have connected alpha decreases in these brain regions to increased activation [52], indicating that hyperactivation occurs during memory maintenance in HIV-infected patients. Taken together, the magnetoencephalography and BOLD findings support our hypothesis that greater frontal-lobe cortical thickness in HIV-infected patients with working memory deficits may be related to neuronal damage by inflammation, as well as activation of brain reserve [53], even if this is unable to achieve normal performance.

The mechanisms involved in HIV brain injury are not fully understood. To assess the participation of the inflammatory process in working memory–related structures, Ernst et al. [54] studied MR spectroscopic and fMRI data from patients with HIV and mild neurocognitive impairment; they demonstrated that increased concentrations of the glial markers Cho, Mi, and Cr in the frontal white matter and basal ganglia were associated with increased BOLD activation during working memory tasks. These findings suggest that working memory deficits in HIV-infected patients are modulated by inflammation in the white matter and basal ganglia. Although we have not studied the basal ganglia and brain spectroscopy, these results are in agreement with our findings, since the brain inflammation caused by the infection may contribute to a greater cortical thickness.

The pathophysiological mechanism of greater cortical thickness in patients with working memory deficit compared with those without such deficit may reflect inflammatory response and/or increased synaptic contacts related to the damage caused by HIV infection, which could contribute to the observed variation in cortical thickness according to cognitive dysfunction stage in HIV-infected patients. Subtle cerebral reorganization reflecting the inherent plasticity of the brain may occur concomitantly with the tissue reduction described in the late stages of AIDS dementia. The greater cortical thickness could be due to inflammation, greater arborization per neuron, increased regional vasculature, or increased glial volume. The methods employed do not distinguish between these possibilities.

Although we did not use a permutation testing and choose a cluster-forming threshold of 1.3 (*p* < .05), we assessed the cortical thickness of the participants, which is less susceptible to false positive rates for surface-based group analysis, compared to volume and area assessments [55]. The two large clusters found in our cortical thickness analysis are more likely to be significant, and true positive. As seen by Greve et al. [55] larger significant clusters of cortical thickness differences can still be significant at the .05 level. The presented study has some limitations. Although our sample was as large as those in most previous studies, it was still relatively small, and the results should be replicated in independent samples. In addition, we could not assess differences between treated and untreated patients, as all patients were receiving HAART. Given the cross-sectional design of the study, we could not examine treatment effects on longitudinal infection-related changes in cortical thickness. However, all HIV-infected patients participating in this study were neurologically asymptomatic, with no significant difference in the duration of known HIV infection, CD4 count, age, education, or sex, and we were able to find significant brain changes in HIV-infected patients with working memory deficit.

Another limitation is that the study design does not include a group of HIV-negative controls. HIV-infected patients formed the two groups studied and their division occurred due to the presence or absence of working memory deficit. The lack of a HIV-negative control group does not allow us to understand whether the cortical thickness of the group with working memory deficit is increased compared to healthy controls or whether the group without working memory deficit could have areas of reduced cortical thickness. Therefore, the interpretation of this study should only be restricted to comparisons between HIV-infected patients, and it is not possible to extrapolate the results to comparisons between HIV-positive patients and healthy controls.

In conclusion, HIV-infected patients with working memory deficit have regions of greater cortical thickness in the left frontal cortex relative to HIV-infected patients with no such deficit. These findings may reflect the effects of HIV-related damage on working memory and may provide insight into the neurobiology of HIV-related brain injury.

## References

[1] Letendre SL, Ellis RJ, Everall I, Ances B, Bharti A, McCutchan JA. Neurologic complications of HIV disease and their treatment. Top HIV Med. 2009;17:46–56. 19401607 PMC3065886

[2] Tozzi V, Balestra P, Bellagamba R, Corpolongo A, Salvatori MF, Visco-Comandini U, et al. Persistence of neuropsychologic deficits despite long-term highly active antiretroviral therapy in patients with HIV- related neurocognitive impairment: prevalence and risk factors. J Acquir Immune Defic Syndr. 2007;45:174–82. doi: 10.1097/QAI.0b013e318042e1ee 17356465

[3] Antinori A, Arendt G, Becker JT, Brew JT, Byrd DA, Cherner M, et al. Updated research nosology for HIV-associated neurocognitive disorders. Neurology. 2007;69:1789–99. doi: 10.1212/01.WNL.0000287431.88658.8b 17914061 PMC4472366

[4] Clifford DB, Ances BM. HIV-associated neurocognitive disorder. Lancet infect Dis. 2013;13:976–86. doi: 10.1016/S1473-3099(13)70269-X 24156898 PMC4108270

[5] Stout JC, Salmon DP, Butters N, Taylor M, Peavy G, Heindel WC, et al. Decline in working memory associated with HIV infection. Psychol Med. 1995;25:1221–32. doi: 10.1017/s0033291700033195 8637952

[6] Woods SP, Moore DJ, Weber E, Grant I. Cognitive neuropsychology of HIV-associated neurocognitive disorders. Neuropsychol Rev. 2009;19:152–68. doi: 10.1007/s11065-009-9102-5 19462243 PMC2690857

[7] Heaton RK, Franklin DR, Ellis RJ, McCutchan JA, Letendre SL, Leblanc S, et al. HIV-associated neurocognitive disorders before and during the era of combination antiretroviral therapy: differences in rates, nature, and predictors. J Neurovirol. 2011;17:3–16. doi: 10.1007/s13365-010-0006-1 21174240 PMC3032197

[8] Bassel C, Rourke SB, Halman MH, Smith ML. Working memory performance predicts subjective cognitive complaints in HIV infection. Neuropsychology. 2002;16:400–10. doi: 10.1037//0894-4105.16.3.400 12146687

[9] Heaton RK, Marcotte TD, Mindt MR, Sadek J, Moore DJ, Bentley H, et al. The impact of HIV-associated neuropsychological impairment on everyday functioning. J Int Neuropsychol Soc. 2004;10:317–31. doi: 10.1017/S1355617704102130 15147590

[10] Power C, Boisse L, Rourke S, Gill MJ. NeuroAIDS: an evolving epidemic. Can J Neurol Sci. 2009;36:285–95. doi: 10.1017/s0317167100007009 19534327

[11] Becker JT, Sanders J, Madsen SK, Ragin A, Kingsley L, Maruca V, et al. Subcortical brain atrophy persists even in HAART-regulated HIV disease. Brain Imaging Behav. 2011;5:77–85. doi: 10.1007/s11682-011-9113-8 21264551 PMC3082694

[12] Küper M, Rabe K, Esser S, Gizewski ER, Husstedt IW, Maschke M, et al. Structural gray and white matter changes in patients with HIV. J Neurol. 2011;258:1066–75. doi: 10.1007/s00415-010-5883-y 21207051

[13] Thompson PM, Dutton RA, Hayashi KM, Toga AW, Lopez OL, Aizenstein HJ, et al. Thinning of the cerebral cortex visualized in HIV/AIDS reflects CD4+ T lymphocyte decline. Proc Natl Acad Sci USA. 2005;102:15647–52. doi: 10.1073/pnas.0502548102 16227428 PMC1266080

[14] Kallianpur KJ, Kirk GR, Sailasuta N, Valcour V, Shiramizu B, Nakamoto BK, et al. Regional cortical thinning associated with detectable levels of HIV DNA. Cereb Cortex. 2012;22:2065–75. doi: 10.1093/cercor/bhr285 22016479 PMC3412442

[15] Becker JT, Maruca V, Kingsley LA, Sanders JM, Alger JR, Barker PB, et al. Factors affecting brain structure in men with HIV disease in the post-HAART era. Neuroradiology. 2012;54:113–21. doi: 10.1007/s00234-011-0854-2 21424708 PMC3154580

[16] Kato T, Yoshihara Y, Watanabe D, Fukumoto M, Wada K, Nakakura T, et al. Neurocognitive impairment and gray matter volume reduction in HIV-infected patients. J Neurovirol. 2020;26:590–601. doi: 10.1007/s13365-020-00865-w 32572834

[17] Ances AM, Ortega M, Vaida F, Heaps J, Paul R. Independent effects of HIV, aging, and HAART on brain volumetric measures. J Acquir Immune Defic Syndr. 2012;59:469–77. doi: 10.1097/QAI.0b013e318249db17 22269799 PMC3302928

[18] Figueiredo VLM, Nascimento E. [Performance in the two tasks of the Digits subtest of WISC-III and WAIS-III]. Psicologia: Teoria e Pesquisa. 2007;23:313–8. Portuguese.

[19] Wechsler D. Wechsler Adult Intelligence Scale, 3rd ed. San Antonio: The Psychological Corporation; 1997.

[20] Saxton J, Snitz BE, Lopez OL, Ives DG, Dunn LO, Fitzpatrick A, et al. Functional and cognitive criteria produce different rates of mild cognitive impairment and conversion to dementia. J Neurol Neurosurg Psychiatry. 2009;80:737–43. doi: 10.1136/jnnp.2008.160705 19279031 PMC2698042

[21] Hill BD, Elliott EM, Shelton JT, Pella RD, O’Jile JR, Gouvier WD. Can we improve the clinical assessment of working memory? An evaluation of the Wechsler Adult Intelligence Scale-Third Edition using a working memory criterion construct. J Clin Exp Neuropsychol. 2010;32:315–23. doi: 10.1080/13803390903032529 19657913 PMC2854874

[22] Cunha JA, Trentini CM, Argimon I. [Brazilian adaptation and standardization of the Wisconsin Card Classification Test Manual]. São Paulo: Casa do Psicólogo; 2005. Portuguese.

[23] Fischl B, Dale AM. Measuring the thickness of the human cerebral cortex from magnetic resonance images. Proc Natl Acad Sci USA. 2000;97:11050–5. doi: 10.1073/pnas.200033797 10984517 PMC27146

[24] Desikan RS, Ségonne F, Fischl B, Quinn BT, Dickerson BC, Blacker D, et al. An automated labeling system for subdividing the human cerebral cortex on MRI scans into gyral based regions of interest. Neuroimage. 2006;31:968–80. doi: 10.1016/j.neuroimage.2006.01.021 16530430

[25] Hagler DJ Jr, Saygin AP, Sereno MI. Smoothing and cluster thresholding for cortical surface-based group analysis of fMRI data. Neuroimage. 2006;33:1093–103. doi: 10.1016/j.neuroimage.2006.07.036 17011792 PMC1785301

[26] Adle-Biassette H, Chrétien F, Wingertsmann L, Héry C, Ereau T, Scaravilli F, et al. Neuronal apoptosis does not correlate with dementia in HIV infection but is related to microglial activation and axonal damage. Neuropathol Appl Neurobiol. 1999;25:123–33. doi: 10.1046/j.1365-2990.1999.00167.x 10216000

[27] Scaravilli F, Bazille C, Gray F. Neuropathologic contributions to understanding AIDS and the central nervous system. Brain Pathol. 2007;17:197–208. doi: 10.1111/j.1750-3639.2007.00047.x 17388950 PMC8095617

[28] Gongvatana A, Woods SP, Taylor MJ, Vigil O, Grant I, HNRC Group. Semantic clustering inefficiency in HIV-associated dementia. J Neuropsychiatry Clin Neurosci. 2007;19:36–42. doi: 10.1176/jnp.2007.19.1.36 17308225

[29] Walker KA, Brown GG. HIV-associated executive dysfunction in the era of modern antiretroviral therapy: a systematic review and meta-analysis. J Clin Exp Neuropsychol. 2018;40:357–76. doi: 10.1080/13803395.2017.1349879 28689493 PMC6164174

[30] Chang L, Lohaugen GC, Andres T, Jiang CS, Douet V, Tanizaki N, et al. Adaptive working memory training improved brain function in human immunodeficiency virus-seropositive patients. Ann Neurol. 2017;81:17–34. doi: 10.1002/ana.24805 27761943 PMC5299494

[31] Khan ZU, Muly EC. Molecular mechanisms of working memory. Behav Brain Res. 2011;219:329–41. doi: 10.1016/j.bbr.2010.12.039 21232555

[32] Smith AB, Smirniotopoulos JG, Rushing EJ. From the archives of the AFIP: central nervous system infections associated with human immunodeficiency virus infection-radiologic-pathologic correlation. RadioGraphics. 2008;28:2033–58. doi: 10.1148/rg.287085135 19001657

[33] Corrêa DG, Zimmermann N, Netto TM, Tukamoto G, Ventura N, Leite SCB, et al. Regional Cerebral Gray Matter Volume in HIV-Positive Patients with Executive Function Deficits. J Neuroimaging. 2016;26:450–7. doi: 10.1111/jon.12327 26780881

[34] Pareto D, Sastre-Garriga J, Auger C, Vives-Gilabert Y, Delgado J, Tintoré M, et al. Juxtacortical lesions and cortical thinning in multiple sclerosis. Am J Neuroradiol. 2015;36:2270–6. doi: 10.3174/ajnr.A4485 26450537 PMC7964289

[35] Hardan AY, Muddasani S, Vemulapalli M, Keshavan MS, Minshew NJ. An MRI study of increased cortical thickness in autism. Am J Psychiatry. 2006;163:1290–2. doi: 10.1176/ajp.2006.163.7.1290 16816240 PMC1509104

[36] Wei G, Zhang Y, Jiang T, Luo J. Increased cortical thickness in sports experts: a comparison of diving players with the controls. PLoS One. 2011;6:e17112. doi: 10.1371/journal.pone.0017112 21359177 PMC3040218

[37] Macey PM, Kheirandish-Gozal L, Prasad JP, Ma RA, Kumar R, Philby MF, et al. Altered regional brain cortical thickness in pediatric obstructive sleep apnea. Front Neurol. 2018;22;9:4. doi: 10.3389/fneur.2018.00004 29403430 PMC5786747

[38] Fortea J, Sala-Llonch R, Bartrés-Faz D, Bosch B, Lladó A, Bargalló N, et al. Increased cortical thickness and caudate volume precede atrophy in PSEN1 mutation carriers. J Alzheimers Dis. 2010;22:909–22. doi: 10.3233/JAD-2010-100678 20858974

[39] Espeseth T, Westlye LT, Fjell AM, Walhovd KB, Rootwelt H, Reinvang I. Accelerated age-related cortical thinning in healthy carriers of apolipoprotein E epsilon 4. Neurobiol Aging. 2008;29:329–40. doi: 10.1016/j.neurobiolaging.2006.10.030 17161508

[40] Espeseth T, Westlye LT, Walhovd KB, Fjell AM, Endestad T, Rootwelt H, et al. Apolipoprotein E ε4-related thickening of the cerebral cortex modulates selective attention. Neurobiol Aging. 2012;33:304–22. doi: 10.1016/j.neurobiolaging.2009.12.027 20382449

[41] Riudavets MA, Iacono D, Resnick SM, O’Brien R, Zonderman AB, Martin LJ, et al. Resistance to Alzheimer’s pathology is associated with nuclear hypertrophy in neurons. Neurobiol Aging. 2007;28:1484–92. doi: 10.1016/j.neurobiolaging.2007.05.005 17599696 PMC2694127

[42] Iacono D, Markesbery WR, Gross M, Pletnikova O, Rudow G, Zandi P, et al. The Nun study: clinically silent AD, neuronal hypertrophy, and linguistic skills in early life. Neurology. 2009;73:665–73. doi: 10.1212/WNL.0b013e3181b01077 19587326 PMC2734290

[43] West MJ, Bach G, Søderman A, Jensen JL. Synaptic contact number and size in stratum radiatum CA1 of APP/PS1DeltaE9 transgenic mice. Neurobiol Aging. 2009;30:1756–76. doi: 10.1016/j.neurobiolaging.2008.01.009 18336954

[44] Oh ES, Savonenko AV, King JF, Fangmark Tucker SM, Rudow GL, Xu G, et al. Amyloid precursor protein increases cortical neuron size in transgenic mice. Neurobiol Aging. 2009;30:1238–44. doi: 10.1016/j.neurobiolaging.2007.12.024 18304698 PMC2796369

[45] Paulsen JS, Magnotta VA, Mikos AE, Paulson HL, Penziner E, Andreasen NC, et al. Brain structure in preclinical Huntington’s disease. Biol Psychiatry. 2006;59:57–63. doi: 10.1016/j.biopsych.2005.06.003 16112655

[46] Hakkers CS, Arends JE, Barth RE, Du Plessis S, Hoepelman AI, Vink M. Review of functional MRI in HIV: effects of aging and medication. J Neurovirol. 2017;23:20–32. doi: 10.1007/s13365-016-0483-y 27718211 PMC5329077

[47] Chang L, Speck O, Miller EN, Braun J, Jovicich J, Koch C, et al. Neural correlates of attention and working memory deficits in HIV patients. Neurology. 2001;57:1001–7. doi: 10.1212/wnl.57.6.1001 11571324

[48] Barker PB, Lee RR, McArthur JC. AIDS dementia complex: evaluation with proton MR spectroscopic imaging. Radiology. 1995;195:58–64. doi: 10.1148/radiology.195.1.7892496 7892496

[49] Kure K, Weidenheim KM, Lyman WD, Dickson DW. Morphology and distribution of HIV-1 gp41-positive microglia in sub-acute AIDS encephalitis. ACTA Neuropathol. 1990;80:393–400. doi: 10.1007/BF00307693 2239151

[50] Vaidya CJ, Austin G, Kirkorian G, Ridlehuber HW, Desmond JE, Glover GH, et al. Selective effects of methylphenidate in attention deficit hyperactivity disorder: a functional magnetic resonance study. Proc Natl Acad Sci USA. 1998;95:14,494–9. doi: 10.1073/pnas.95.24.14494 9826728 PMC24401

[51] Wilson TW, Proskovec AL, Heinrichs-Graham E, O’Neill J, Robertson KR, Fox HS, et al. Aberrant Neuronal Dynamics during Working Memory Operations in the Aging HIV-Infected Brain. Sci Rep. 2017;7:41568. doi: 10.1038/srep41568 28155864 PMC5290733

[52] Murta T, Leite M, Carmichael DW, Figueiredo P, Lemieux L. Electrophysiological correlates of the BOLD signal for EEG-informed fMRI. Hum Brain Mapp. 2015;36:391–414. doi: 10.1002/hbm.22623 25277370 PMC4280889

[53] Cabeza R, Albert M, Belleville S, Craik FIM, Duarte A, Grady CL, et al. Maintenance, reserve and compensation: the cognitive neuroscience of healthy ageing. Nat Rev Neurosci. 2018;19:701–10. doi: 10.1038/s41583-018-0068-2 30305711 PMC6472256

[54] Ernst T, Chang L, Arnold S. Increased glial metabolites predict increased working memory network activation in HIV brain injury. Neuroimage. 2003;19:1686–93. doi: 10.1016/s1053-8119(03)00232-5 12948723

[55] Greve DN, Fischl B. False positive rates in surface-based anatomical analysis. Neuroimage. 2018;171:6–14. doi: 10.1016/j.neuroimage.2017.12.072 29288131 PMC5857431

